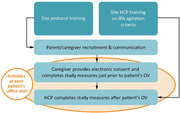# Study Protocol: Quantitative evaluation of The Agitation in Alzheimer’s Screener for Caregivers AASC™, a novel tool for improving recognition of agitation in Alzheimer’s dementia

**DOI:** 10.1002/alz.094799

**Published:** 2025-01-09

**Authors:** Carolyn K Clevenger, Malaak Brubaker, William C Jackson, Jared Stroud, Sue Peschin, Sheri Fehnel, T. Michelle Brown, Emily Bratlee‐Whitaker, Iwona Bucior, Mehul Patel, George T Grossberg

**Affiliations:** ^1^ Emory University, Atlanta, GA USA; ^2^ Otsuka Pharmaceutical Development & Commercialization Inc., Princeton, NJ USA; ^3^ University of Tennessee College of Medicine, Memphis, TN USA; ^4^ OhioHealth, Columbus, OH USA; ^5^ Alliance of Aging Research, Washington, DC USA; ^6^ RTI Health Solutions, Research Triangle Park, NC USA; ^7^ Otsuka Pharmaceutical, Princeton, NJ USA; ^8^ National Research Council, Montreal, QC Canada; ^9^ St. Louis University, St. Louis, MO USA

## Abstract

**Background:**

Agitation, manifesting as aggressive and non‐aggressive behaviors, is one of the most common neuropsychiatric symptoms in Alzheimer’s dementia, presenting in approximately half of all patients. Despite the high prevalence, recognition of agitation in Alzheimer’s dementia (AAD) remains a challenge that impacts timely diagnosis and treatment. The International Psychogeriatric Association (IPA) established a new standard definition of agitation in cognitive disorders, which provides guidance for advancing recognition and improving patient care. The Agitation in Alzheimer’s Screener for Caregivers (AASC™), an easy‐to‐use and pragmatic tool, was developed based on IPA criteria to support caregivers and healthcare professionals (HCPs) in recognizing AAD, thereby facilitating caregiver‐HCP discussions and supporting timely treatment planning.

**Method:**

The AASC™ was developed and qualitatively evaluated through a rigorous, iterative process involving clinical experts, patients, and caregivers. For quantitative validation, this prospective, multisite, single‐visit observational study will calculate predictive metrics for the identification of agitation by comparing responses on the AASC™, completed by caregivers, to HCPs judgement based on implementation of the IPA criteria (**Figure 1**). Community‐dwelling patients must have a recorded diagnosis of Alzheimer’s dementia; caregivers must be aged 18‐85 years, provide patient care ≥10 hours/week, and attend the office visit. Data will be collected and analyzed in 2 parts: an interim analysis (n = 50 dyads) will determine the rate of agreement (yes/no), and a final analysis (n = 150 dyads) will determine sensitivity and specificity of the AASC™. Caregiver‐reported items include caregiver and patient demographics and AASC™ responses. HCPs will complete assessments for the presence of IPA‐defined agitation and the severity of Alzheimer’s dementia.

**Result:**

Results will include: descriptive statistics, the percentage agreement and Cohen’s kappa coefficient characterizing the interrater reliability between the caregiver‐completed AASC™ and IPA‐based HCP decision (part 1), sensitivity, specificity, positive and negative predictive values of the AASC™ against the HCP decision, goodness of fit metrics (e.g., concordance), and receiver operating characteristic curves (part 2).

**Conclusion:**

The AASC™ was developed following a rigorous process established to support its medical credibility and implementation in clinical practice. Results from this study will quantitatively validate the AASC™, leveraging caregiver observations to aid in facilitating earlier identification and treatment of AAD.